# SyBLaRS: A web service for laying out, rendering and mining biological maps in SBGN, SBML and more

**DOI:** 10.1371/journal.pcbi.1010635

**Published:** 2022-11-14

**Authors:** Hasan Balci, Ugur Dogrusoz, Yusuf Ziya Ozgul, Perman Atayev

**Affiliations:** 1 i-Vis Research Lab, Computer Engineering Department, Bilkent University, Ankara, Turkey; 2 Odoo S.A., Chaussée de Namur, Belgium; University of Connecticut School of Medicine, UNITED STATES

## Abstract

Visualization is a key recurring requirement for effective analysis of relational data. Biology is no exception. It is imperative to annotate and render biological models in standard, widely accepted formats. Finding graph-theoretical properties of pathways as well as identifying certain paths or subgraphs of interest in a pathway are also essential for effective analysis of pathway data. Given the size of available biological pathway data nowadays, automatic layout is crucial in understanding the graphical representations of such data. Even though there are many available software tools that support graphical display of biological pathways in various formats, there is none available as a service for on-demand or batch processing of biological pathways for automatic layout, customized rendering and mining paths or subgraphs of interest. In addition, there are many tools with fine rendering capabilities lacking decent automatic layout support.

To fill this void, we developed a web service named SyBLaRS (Systems Biology Layout and Rendering Service) for automatic layout of biological data in various standard formats as well as construction of customized images in both raster image and scalable vector formats of these maps. Some of the supported standards are more generic such as GraphML and JSON, whereas others are specialized to biology such as SBGNML (The Systems Biology Graphical Notation Markup Language) and SBML (The Systems Biology Markup Language). In addition, SyBLaRS supports calculation and highlighting of a number of well-known graph-theoretical properties as well as some novel graph algorithms turning a specified set of objects of interest to a minimal pathway of interest.

We demonstrate that SyBLaRS can be used both as an offline layout and rendering service to construct customized and annotated pictures of pathway models and as an online service to provide layout and rendering capabilities for systems biology software tools.

SyBLaRS is open source and publicly available on GitHub and freely distributed under the MIT license. In addition, a sample deployment is available here for public consumption.

This is a *PLOS Computational Biology* Software paper.

## Introduction

With help from widely accepted standard file formats such as SBGNML [1] and SBML [2] and numerous pathway databases such as Pathway Commons [3], WikiPathways [4], and Reactome [5], a vast amount of biological process data is now available to the scientific community in a format suitable for computation.

Visualization of relational data via maps/pathways is compelling in all domains including biology as it is believed to bring out patterns, broad relationships and emerging trends in data, for an insightful analysis [6]. In visualizing relational information, a good layout of objects and their relations is vital since a poor layout will confuse the user, and an average user is expected to spend up to 25 percent of their time on manual layout adjustments [7].

Many web-based [8] and standalone tools [9], [10], [11] have been built for visual construction and analysis of biological pathways. Some of these tools have strong automatic layout capabilities while others rely on the user manually adjusting the map layout in need of an automatic layout service. In addition, while many prominent pathway databases such as Reactome [5] and BioModels [12] make their content available in widely used standard file formats, a visual display of such pathway data is either not available or done laboriously in a manual fashion, creating constant work for the team as new pathways are curated. Furthermore, currently available tools lack support for calculation and proper highlighting of graph-theoretical properties of pathways as well as operations to mine large pathways to find and show parts of pathways that are of particular interest to a user.

A similar past effort providing such a layout service [13] works only for input BioPAX models [14], lacking support for input in newer standards such as SBGNML and SBML, with little room for configuration of both the layout operation and the image construction and without a mining facility.

**Fig 1:**
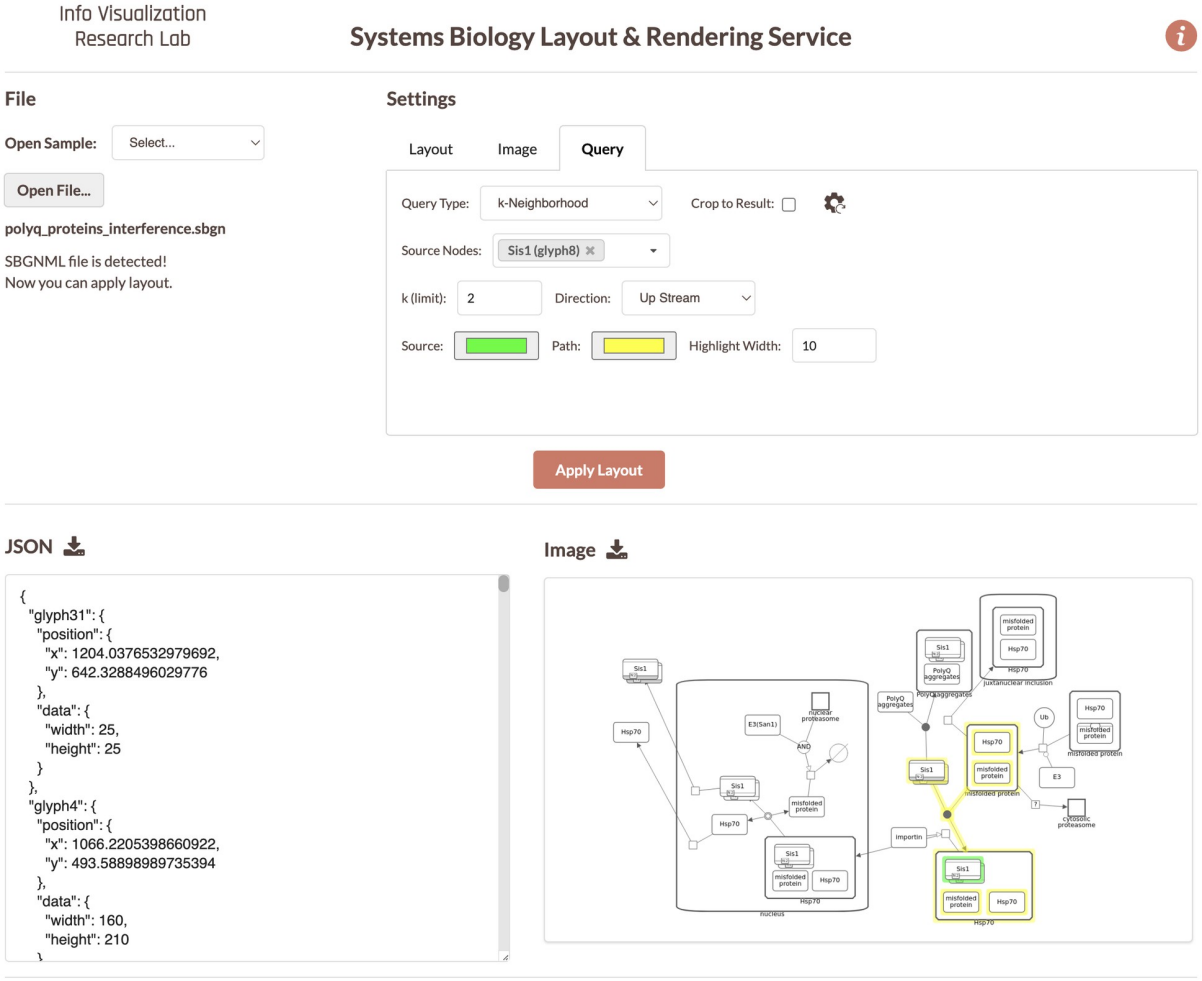
A screenshot from the user interface of a sample public deployment of the SyBLaRS web service, where 2-level upstream neighborhood of a protein is highlighted.

With this paper, we introduce a web service named Systems Biology Layout & Rendering Service (SyBLaRS) with the aim to fill the void for programmatically generating graphical representations of pathways while optionally highlighting paths or sub-pathways of interest and automatically laying them out (Fig 1). This also facilitates usage as a layout service by third-party visual pathway analysis software.

SyBLaRS was built on Cytoscape.js [15] graph visualization library with the following main use cases in mind:

- Create an image of provided biological maps (already with layout information) in popular standard formats,
- Lay out the provided map in specified layout style chosen from many available ones and return the map with layout information,
- Both lay out the provided map in specified layout style and create an image of it as raster image or scalable vector image formats, and
- Optionally highlight a path or a sub-pathway (nodes, edges, or paths of interest) based on the results of a graph-based query.

## Materials and methods

SyBLaRS accommodates a number of novel methods as well as widely known and used ones on automatic layout of pathways [16, 17], calculating graph-theoretic properties [18] in pathways and mining pathways for subgraphs of interest [19]. The service makes use of Cytoscape.js [20] for some operations in addition to using it as its base rendering facility for constructing images as detailed in the next section.

### Automatic layout algorithms

**Fig 2:**
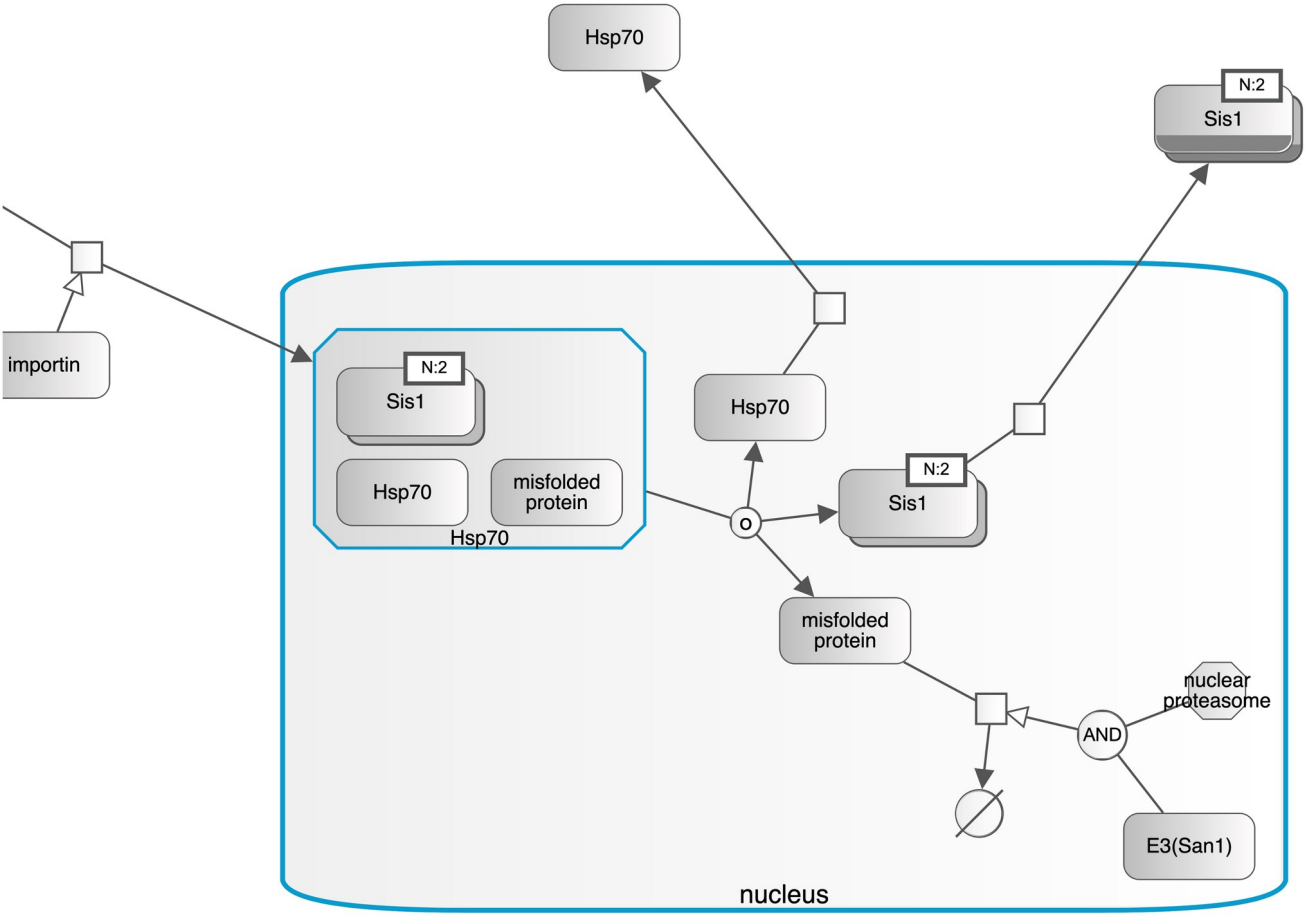
Part of an example pathway where compound structures are used to represent cellular compartments such as nucleus and molecular complexes such as Hsp70 (highlighted in blue in this example).

Even though SyBLaRS supports a wide range of automatic layout algorithms written as Cytoscape.js extensions, of particular interest is fCoSE [16] that supports the compound structures. Such structures are typically depicted as nested drawings in biological pathways representing compartments or molecular complexes (Fig 2). The fCoSE layout algorithm builds on a previous compound spring embedder layout algorithm, making use of the spectral graph drawing technique for producing a quick draft layout. It then applies heuristics where constraints are enforced and compound structures are properly shown. Meanwhile the layout is polished with respect to commonly accepted graph layout criteria.

All available layout algorithms are customizable for one’s specific needs (e.g., ideal edge length and whether or not any available coordinates should be taken into account as opposed to starting from scratch) through the associated API. Similarly, all are suitable for interactive use as they complete in at most a few seconds for pathways of up to several hundred biological entities and similar number of interaction edges. Please refer to the associated GitHub repository for a complete set of options.

### Graph properties and mining algorithms

One can predict the behavior of biological networks using measured graph-theoretical or structural properties of those systems as well as the local rules governing individual objects of the networks [21]. Hence, a researcher might like to analyze a particular pathway making use of its graph-theoretical properties such as betweenness centrality. SyBLaRS enables this through its query API allowing the calculation and display of the following properties:

- Degree centrality,
- Closeness centrality,
- Betweenness centrality, and
- Page rank.

**Fig 3:**
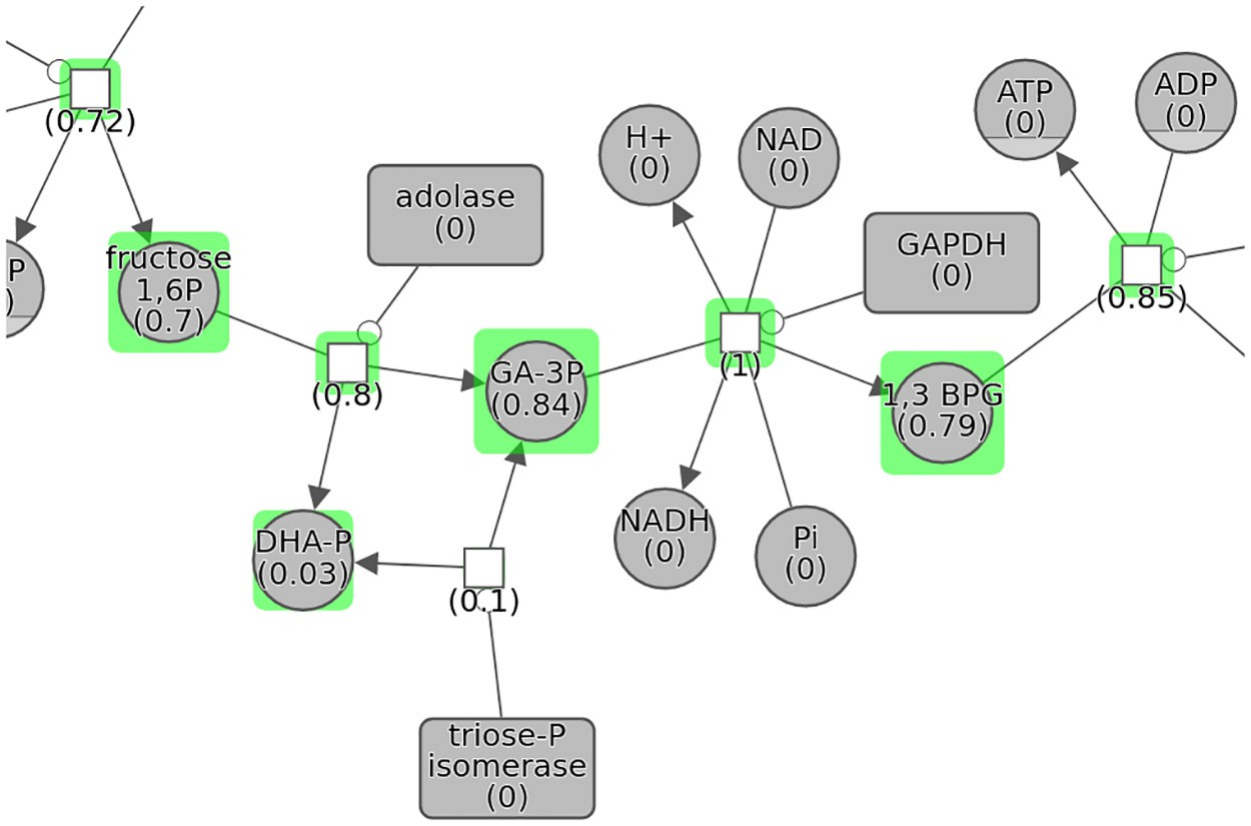
Normalized betweenness centrality values in part of a pathway are shown both as part of node labels (numbers in parentheses) and node highlight thickness (the higher the centrality value, the thicker the highlight thickness).

Such properties are displayed as part of node labels and optionally emphasized through node highlight as exemplified in Fig 3.

Oftentimes one is interested in discovering and analyzing certain types of paths or sub-pathways in a given biological pathway model, especially in large ones. In order to facilitate such queries, the algorithms in [19] were adapted to work with pathways in our supported formats.

#### Shortest paths

SyBLaRS exposes the shortest paths algorithm of Dijkstra as provided by Cytoscape.js library, where the user specifies the source and target nodes and a single path of a shortest length between these nodes are highlighted with the specified color.

The remaining graph mining queries were originally designed in [19] with the aim to extract paths or sub-pathways of interest from a given set of nodes of interest in a pathway database using a non-standard, proprietary notation. We adapted these algorithms to work with the current standard formats.

Even though these algorithms seem like straightforward implementations of well-known graph algorithms such as Dijkstra’s shortest paths [22], they differ from those in the following ways:

- They support compound structures through modified DFS and BFS traversals, where upon reaching a particular compound node or a node within a compound node, we also visit its children or parent/sibling nodes, so as to seamlessly continue the traversal. The user has the option to choose the traversal direction (downstream to follow the edge directions in normal manner, upstream to follow them in the reverse direction and both streams to go in both directions).
- Dijkstra’s algorithm finds one of many potentially available shortest paths from a single dedicated node to another dedicated one, whereas algorithms such as Paths-between and Paths-from-to find *all* such paths between a *group* of source and target nodes. Besides, some of these algorithms allow one to define additional distance. For instance, if the actual shortest path between one source and one target node is 3 and the additional distance is 2, then these algorithms will find all paths of length between 3, 4 or 5.

#### Neighborhood

This is a simple neighborhood query where one can specify a group of nodes, from all of which a parallel compound BFS is started. Any node discovered during these traversals gets added to the result (see Fig 1 for an example).

#### Common stream

**Fig 4:**
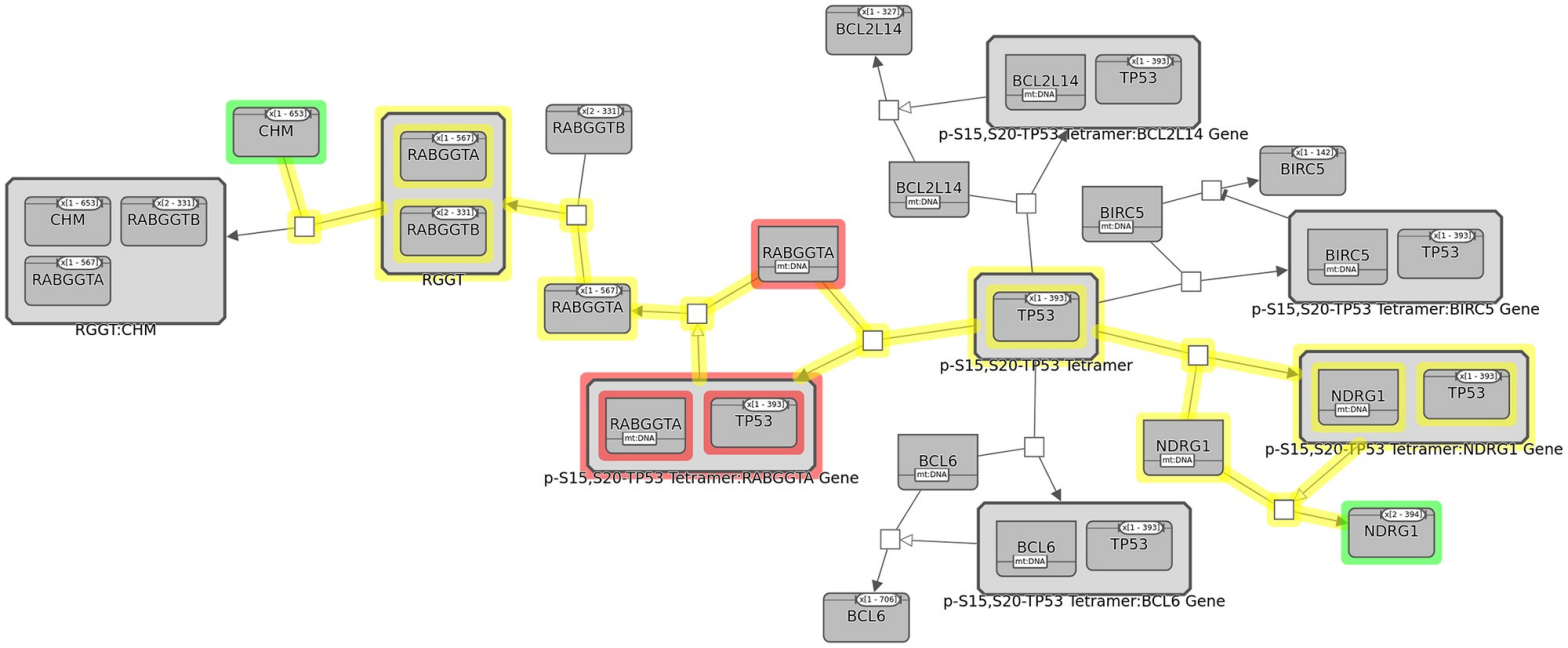
An example of the common stream query where we identify and highlight the common targets/regulators (in red) of two proteins NDRG1 and CHM (in green). Connecting links are also highlighted (in yellow).

This query aims to find those nodes that are common to the up (common regulator), down (common target) or both streams of a specified group of entities. One often wants to know if there is a gene in the upstream of some genes in a pathway, which can provide a causal explanation for the co-regulation (eventually a way to control the associated module) [19]. Or two pathways affecting the same mechanism in an organism might be interesting as it suggests that a specific phenotype can have multiple molecular causes. Finding common targets of signaling proteins might help one develop alternative treatment strategies [23]. Fig 4 shows an example common stream query.

With the latest high-throughput sequencing technologies, one can scan for alterations in large quantities of samples [24]. A problem that appears regularly in high-throughput studies is selection of genes/proteins. One convenient way to determine new genes for sequencing is to find the vicinity of these genes implied in that particular complex disease. These genes that connect two or more of these “usual suspects” within a signaling path are expected to be of more significance for the disease [19]. Following queries address use cases like this.

#### Paths-between

**Fig 5:**
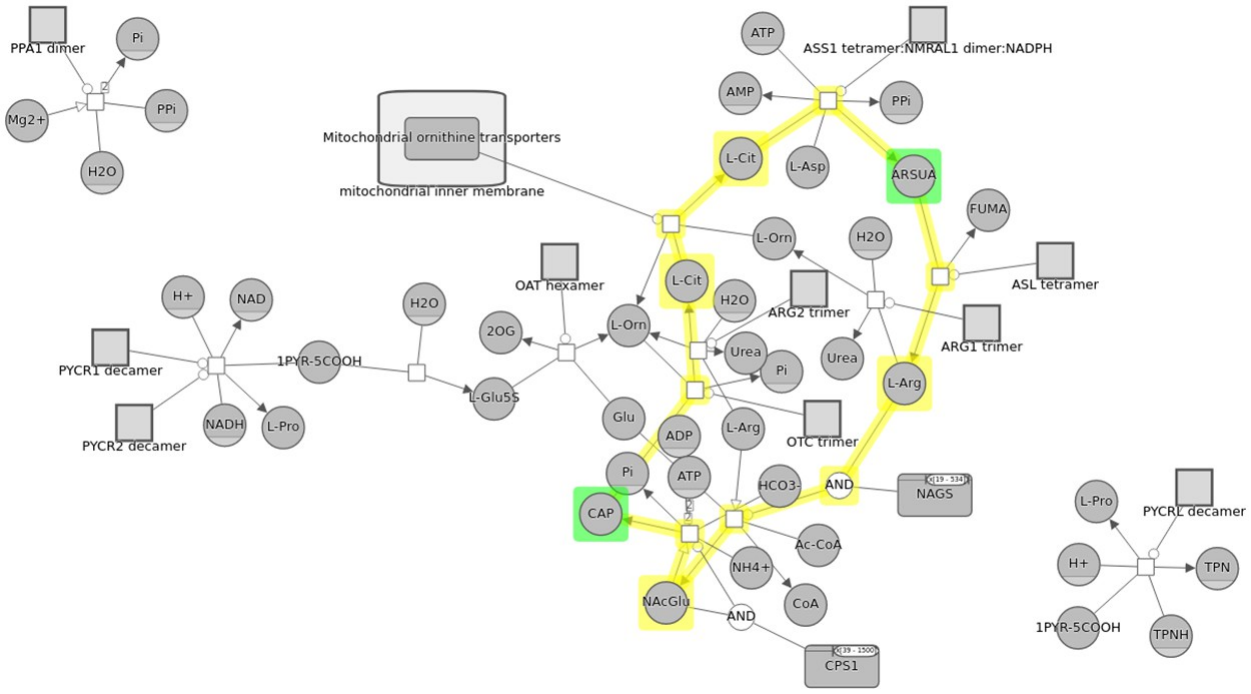
An example paths-between query to find the two distinct directed links between two simple chemicals in the Urea cycle pathway [5] with *k* = 7.

Paths-between query finds a “maximal” pathway comprising all the nodes of interest complemented by the “missing links” among these nodes. Its parameter *k* defines the maximum length of these links. Fig 5 shows an example paths-between query.

#### Paths-from-to

Finding shortest paths between a single or all pairs of vertices in a graph is a well known and commonly used algorithm [22]. This query is a more general one, where the goal is to find *all* shortest paths from a source node set *S* to a target one *T*. The query could be constrained with a maximum length *k* of such paths. Furthermore, another parameter *d* is provided for relaxing the shortest requirement.

Even though traversal based queries discussed above might be quite useful in mining sub-pathways of interest, they should be used with caution in large pathways due to their exponential nature in number of such paths of interest.

### Image construction

**Fig 6:**
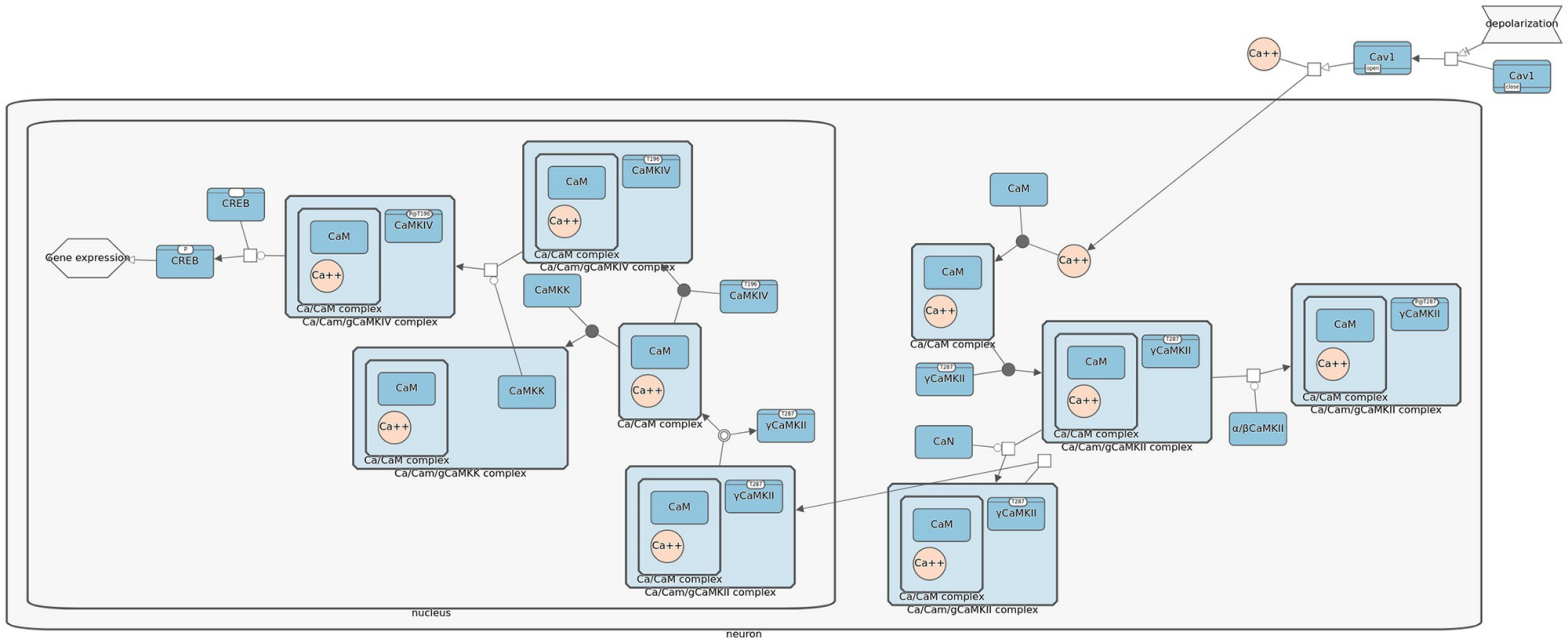
An example pathway image automatically styled and constructed by SyBLaRS using a red-blue color scheme (red for simple chemicals and blue for macromolecules).

Optionally, an image of the provided map is created in the specified format (JPG, PNG or SVG). Construction can be tailored in certain ways such as the image dimensions and the color scheme to be used. With some file formats, a single color is specified and SyBLaRS uses different shades of that color for rendering graph elements of varying types, whereas in some others, there are specific color schemes (e.g., red-blue) to choose from (Fig 6). The user can also specify whether or not the map should have a background of a specified color. Furthermore, when the result of a query is shown, the user may choose to include only the resulting paths or sub-pathways in the image as opposed to the whole pathway. Please refer to the associated GitHub repository for a complete set of options.

## Design and implementation

**Fig 7:**
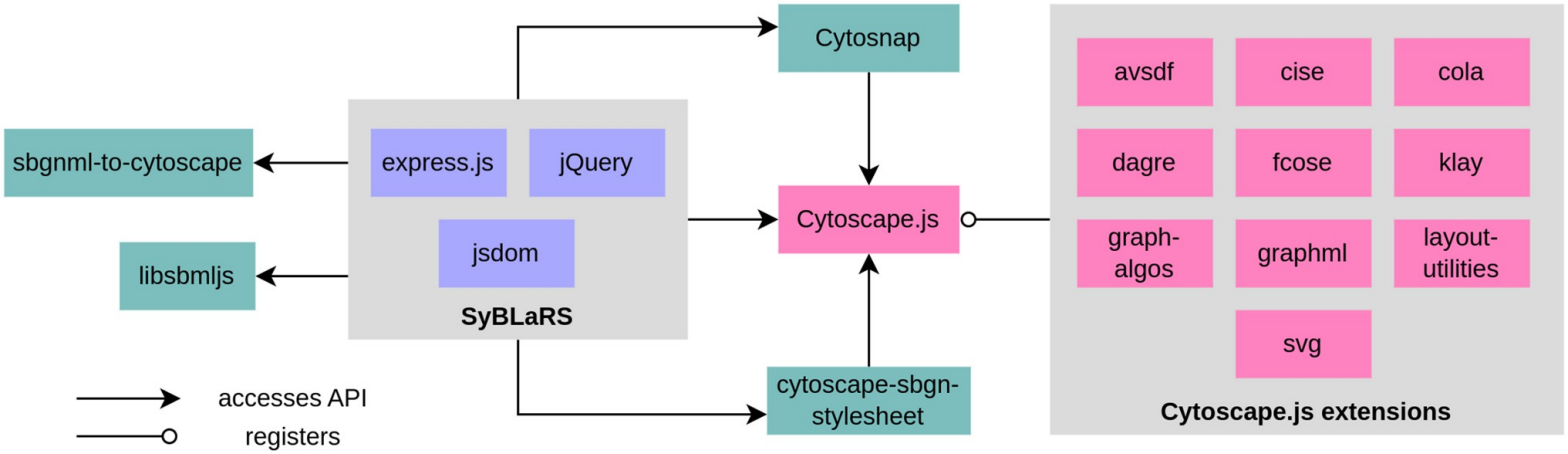
Diagram showing software libraries used for building SyBLaRS.

SyBLaRS uses a package named *cytoscape-sbgn-stylesheet* [25] that provides a stylesheet for the SBGN maps. For other formats, we have designed and implemented our own stylesheets. Certain graph algorithms used to calculate graph-theoretic properties of the pathways come from the Cytoscape.js core, whereas others specialized in finding subgraphs of interest are from the *cytoscape-graph-algos* extension. SyBLaRS’s architecture is sketched in Fig 7. SyBLaRS mainly depends on the Cytoscape.js library to draw graphs and manage graph operations. It uses *sbgnml-to-cytoscape* [26] and *libsbmljs* [27] packages and the *cytoscape-graphml* extension to convert SBGNML, SBML and GraphML files, respectively, into JSON format as accepted by Cytoscape.js. *Cytosnap*, which is a package to render graphs on the server side with Cytoscape.js, is used to apply layout and/or generate images of pathway graphs.

**Fig 8:**
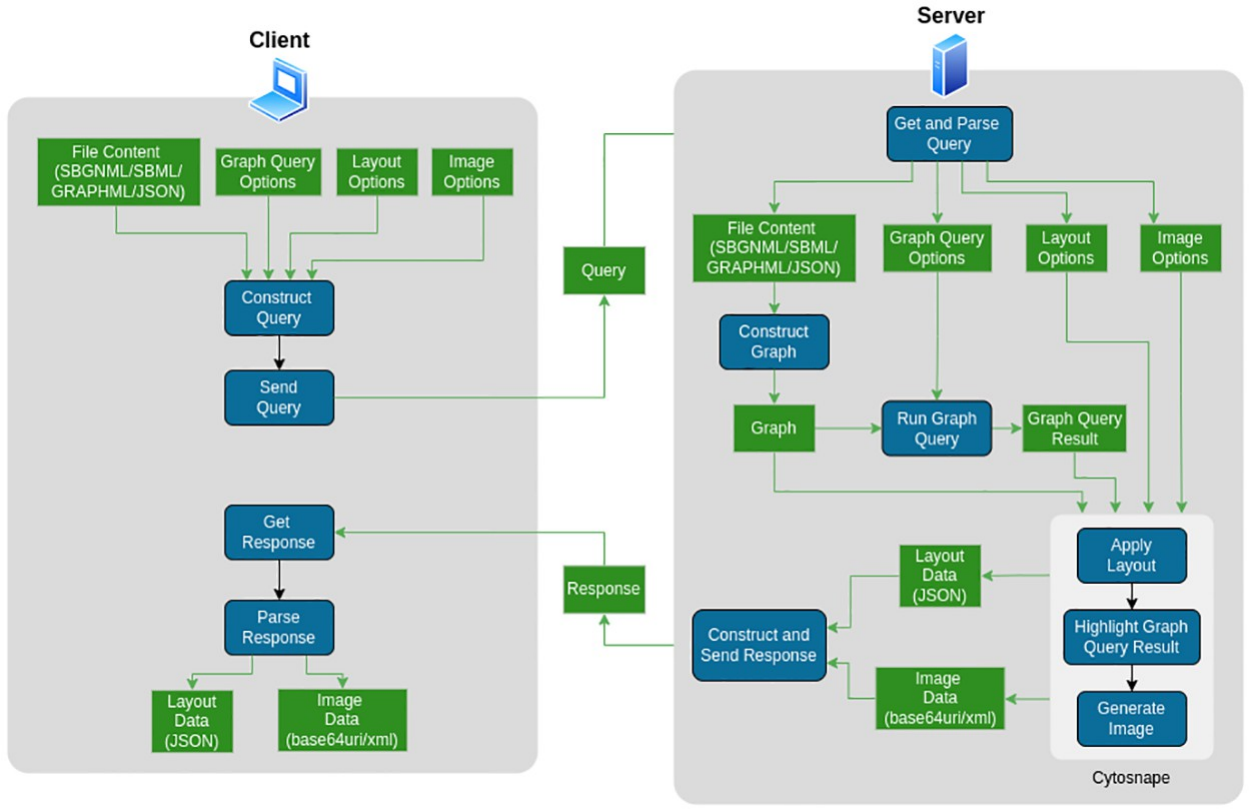
Data (shown as rectangles in green) flow as well as the sequencing of activities (shown as rounded rectangles in blue) both in the client and server sides and across sides of SyBLaRS is shown graphically.

Fig 8 shows the data flow and activity sequencing in SyBLaRS. The user is expected to form a query consisting of the file content and one or more of the layout, image and graph query options and to send it to the server. SyBLaRS first gets the query and parses it to its components. Then the corresponding Cytoscape.js graph is constructed from the file content. In case a graph query is applicable, it gets executed using the provided graph query options. SyBLaRS then transfers the graph, result of the graph query, layout and image options to Cytosnap, which first applies the specified layout, then highlights any query result, finally generating the corresponding image. SyBLaRS constructs a response message comprising the layout and image data, which are the outputs from Cytosnap and sent back to the user. Upon getting this response, the user is expected to parse it to its components and obtain the desired layout and image data.

## Results

Once a service is set up, requests can be made through a web page, like the one used by our sample public deployment, or programmatically in the background without a user interface, say to produce an image of a newly curated pathway as it becomes available. Supported input file formats are SBGNML [1], SBML [2], GraphML [28], and JSON.

Below is an example request to our sample deployment via curl:

curl -X POST -H “Content-Type: text/plain” --data

“request_body” syblars.cs.bilkent.edu.tr/file_format

where, the file_format is one of sbgnml, sbml, graphml and json, and the request_body includes the map content and any layout, image and query options as detailed in the GitHub repository README.

All three operations, performing a layout on the provided map, running a query on it and creating an image of it, are optional. If preferred, the service will return the layout information in JSON format. The format of the optional output of an image of the map is JPG, PNG or SVG.

SyBLaRS has multiple use cases as a service including, but not limited to the following.

### For batch processing

One can use SyBLaRS to quickly generate images for pathway models, where an optional automatic layout can also be performed. In fact, this could be automated in such a way that, in pathway databases where new models emerge periodically, a tool using a SyBLaRS service could construct such images on demand.

**Fig 9:**
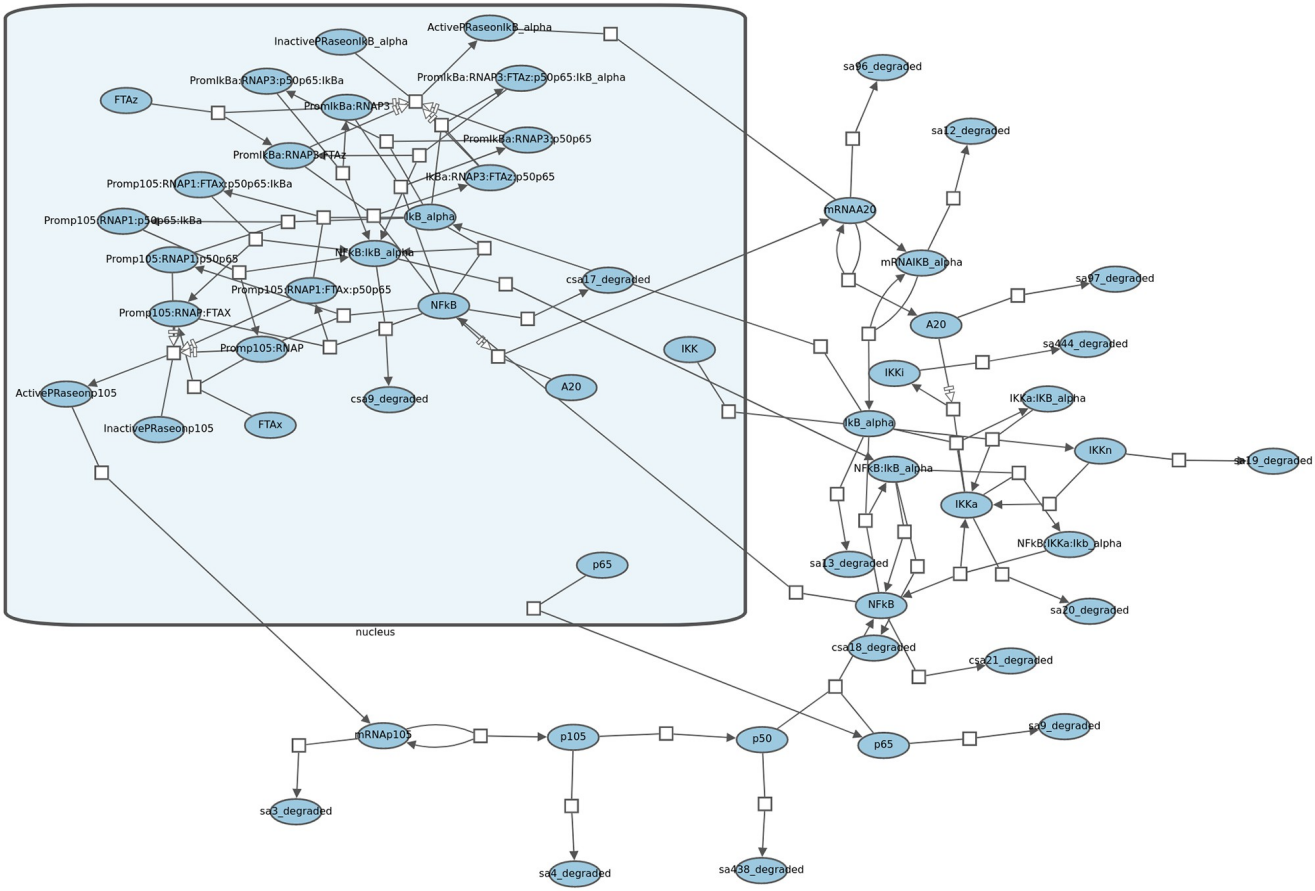
The image generated for one of the pathways in BioModels dataset. (https://www.ebi.ac.uk/biomodels/MODEL7743631122).

We tested this use case for a hundred BioModels pathways [12] that are publicly available. In a matter of minutes, SyBLaRS was able to construct images of BioModels pathways with proper layout. These models and the corresponding images generated by SyBLaRS can be found on GitHub. See Fig 9 for an example of such an image as produced by SyBLaRS.

### As a layout service

SyBLaRS may also be used as a layout service in pathway analysis applications, where the tool has rendering capabilities of its own but not a proper automatic layout facility.

This use case has been tested with Newt [8], a web based pathway editor with advanced layout facilities by simply replacing the existing layout operations with remote ones on a SyBLaRS service. Sample runs proved acceptable in terms of execution time for interactive use.

### Individual use

The publicly available deployment of SyBLaRS may also be used by individuals who would like to highlight certain paths of interest in their pathways or simply construct images of their pathways for inclusion in a document such as a scientific article. With SyBLaRS’s user-friendly and simple graphical user interface, one can easily generate such images without having to install software or learn sophisticated pathway visualization tools.

## Availability and future directions

SyBLaRS can be used both as an offline layout and rendering service to construct customized and annotated pictures of pathway models and as an online service to provide layout and rendering capabilities for systems biology software tools. In addition, it may be used for merely constructing automatically laid out static images of your pathway models with optional support to highlight paths or sub-pathways of your interest.

SyBLaRS is open source and publicly available on GitHub and freely distributed under the MIT license. In addition, a sample deployment is available here for public consumption. The service in this deployment may also be used programmatically from your own application.

As future work, we would like to embed newly calculated layout information in the provided file using the same format as the input, as opposed to returning it separately as JSON. We would also like to extend our query and layout (especially those styles that support Bezier curves for routing relations to produce aesthetically more pleasing layouts) algorithms.

## Supporting information

S1 Video Main capabilities of SyBLaRS demonstrated. This video demonstrates how the public SyBLaRS deployment can be used to perform automatic layout and querying features of the service as well as construction of an image of the provided pathway model.
(MP4)
